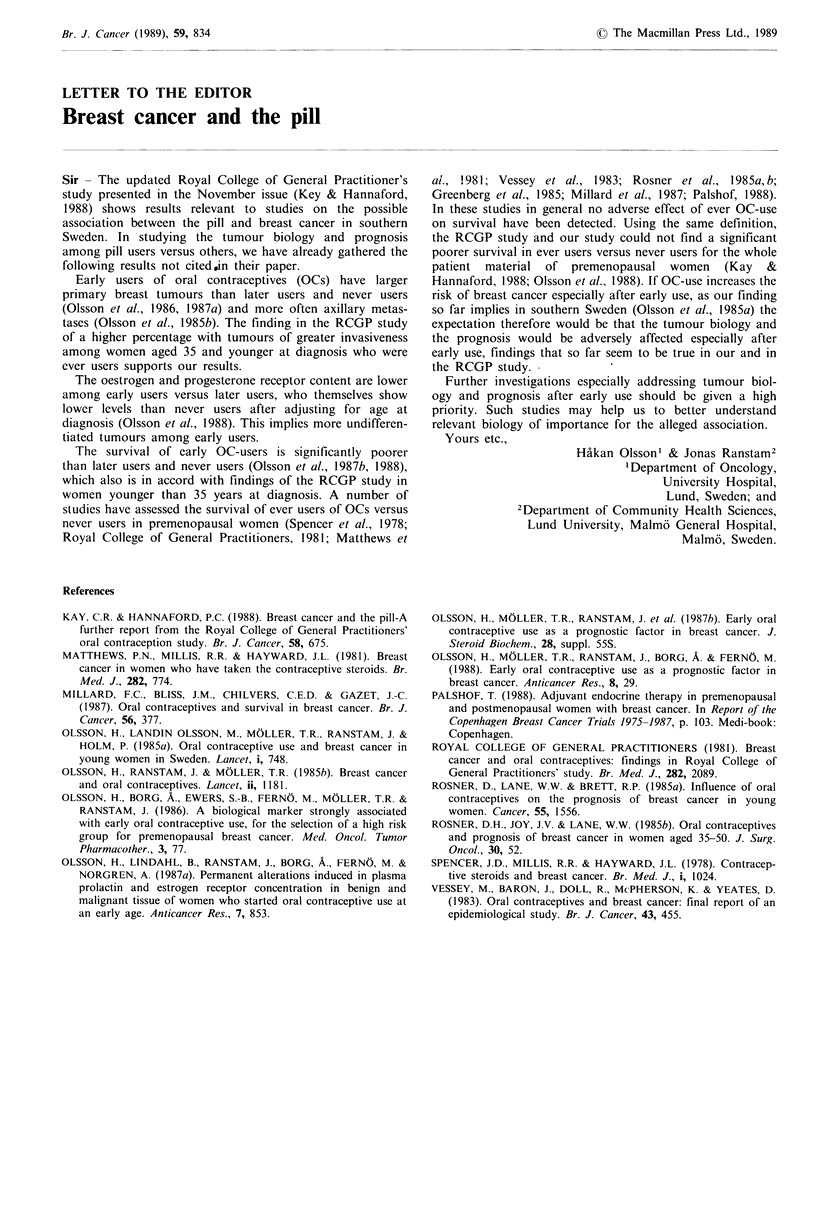# Breast cancer and the pill.

**DOI:** 10.1038/bjc.1989.173

**Published:** 1989-05

**Authors:** H. Olsson, J. Ranstam


*Br. J. Cancer* (1989), **59**, 834
© The Macmillan Press Ltd., 1989

### LETTER TO THE EDITOR

### Breast cancer and the pill

**Sir** – The updated Royal College of General Practitioner's study presented in the November issue (Key & Hannaford, 1988) shows results relevant to studies on the possible association between the pill and breast cancer in southern Sweden. In studying the tumour biology and prognosis among pill users versus others, we have already gathered the following results not cited in their paper.

Early users of oral contraceptives (OCs) have larger primary breast tumours than later users and never users (Olsson *et al.*, 1986, 1987*a*) and more often axillary metastases (Olsson *et al.*, 1985*b*). The finding in the RCGP study of a higher percentage with tumours of greater invasiveness among women aged 35 and younger at diagnosis who were ever users supports our results.

The oestrogen and progesterone receptor content are lower among early users versus later users, who themselves show lower levels than never users after adjusting for age at diagnosis (Olsson *et al.*, 1988). This implies more undifferentiated tumours among early users.

The survival of early OC-users is significantly poorer than later users and never users (Olsson *et al.*, 1987*b*, 1988), which also is in accord with findings of the RCGP study in women younger than 35 years at diagnosis. A number of studies have assessed the survival of ever users of OCs versus never users in premenopausal women (Spencer *et al.*, 1978; Royal College of General Practitioners, 1981; Matthews *et al.*, 1981; Vessey *et al.*, 1983; Rosner *et al.*, 1985*a,b*; Greenberg *et al.*, 1985; Millard *et al.*, 1987; Palshof, 1988). In these studies in general no adverse effect of ever OC-use on survival have been detected. Using the same definition, the RCGP study and our study could not find a significant poorer survival in ever users versus never users for the whole patient material of premenopausal women (Kay & Hannaford, 1988; Olsson *et al.*, 1988). If OC-use increases the risk of breast cancer especially after early use, as our finding so far implies in southern Sweden (Olsson *et al.*, 1985*a*) the expectation therefore would be that the tumour biology and the prognosis would be adversely affected especially after early use, findings that so far seem to be true in our and in the RCGP study.

Further investigations especially addressing tumour biology and prognosis after early use should be given a high priority. Such studies may help us to better understand relevant biology of importance for the alleged association.

Yours etc.,

Håkan Olsson[1] & Jonas Ranstam[2]
[1]Department of Oncology, University Hospital, Lund, Sweden; and
[2]Department of Community Health Sciences, Lund University, Malmö General Hospital, Malmö, Sweden.

### References

KAY, C.R. & HANNAFORD, P.C. (1988). Breast cancer and the pill-A further report from the Royal College of General Practitioners' oral contraception study. *Br. J. Cancer*, **58**, 675.

MATTHEWS, P.N., MILLIS, R.R. & HAYWARD, J.L. (1981). Breast cancer in women who have taken the contraceptive steroids. *Br. Med. J.*, **282**, 774.

MILLARD, F.C., BLISS, J.M., CHILVERS, C.E.D. & GAZET, J.-C. (1987). Oral contraceptives and survival in breast cancer. *Br. J. Cancer*, **56**, 377.

OLSSON, H., LANDIN OLSSON, M., MÖLLER, T.R., RANSTAM, J. & HOLM, P. (1985*a*). Oral contraceptive use and breast cancer in young women in Sweden. *Lancet*, **i**, 748.

OLSSON, H., RANSTAM, J. & MÖLLER, T.R. (1985*b*). Breast cancer and oral contraceptives. *Lancet*, **ii**, 1181.

OLSSON, H., BORG, Å., EWERS, S.-B., FERNÖ, M., MÖLLER, T.R. & RANSTAM, J. (1986). A biological marker strongly associated with early oral contraceptive use, for the selection of a high risk group for premenopausal breast cancer. *Med. Oncol. Tumor Pharmacother.*, **3**, 77.

OLSSON, H., LINDAHL, B., RANSTAM, J., BORG, Å., FERNÖ, M. & NORGREN, A. (1987*a*). Permanent alterations induced in plasma prolactin and estrogen receptor concentration in benign and malignant tissue of women who started oral contraceptive use at an early age. *Anticancer Res.*, **7**, 853.

OLSSON, H., MÖLLER, T.R., RANSTAM, J. *et al.* (1987*b*). Early oral contraceptive use as a prognostic factor in breast cancer. *J. Steroid Biochem.*, **28**, suppl. 55S.

OLSSON, H., MÖLLER, T.R., RANSTAM, J., BORG, Å. & FERNÖ, M. (1988). Early oral contraceptive use as a prognostic factor in breast cancer. *Anticancer Res.*, **8**, 29.

PALSHOF, T. (1988). Adjuvant endocrine therapy in premenopausal and postmenopausal women with breast cancer. In *Report of the Copenhagen Breast Cancer Trials 1975–1987*, p. 103. Medi-book: Copenhagen.

ROYAL COLLEGE OF GENERAL PRACTITIONERS (1981). Breast cancer and oral contraceptives: findings in Royal College of General Practitioners' study. *Br. Med. J.*, **282**, 2089.

ROSNER, D., LANE, W.W. & BRETT, R.P. (1985*a*). Influence of oral contraceptives on the prognosis of breast cancer in young women. *Cancer*, **55**, 1556.

ROSNER, D.H., JOY, J.V. & LANE, W.W. (1985*b*). Oral contraceptives and prognosis of breast cancer in women aged 35–50. *J. Surg. Oncol.*, **30**, 52.

SPENCER, J.D., MILLIS, R.R. & HAYWARD, J.L. (1978). Contraceptive steroids and breast cancer. *Br. Med. J.*, **i**, 1024.

VESSEY, M., BARON, J., DOLL, R., McPHERSON, K. & YEATES, D. (1983). Oral contraceptives and breast cancer: final report of an epidemiological study. *Br. J. Cancer*, **43**, 455.